# ﻿George Gardner’s enigmatic species *Goyaziavillosa* (Gesneriaceae) finally rediscovered

**DOI:** 10.3897/phytokeys.246.125734

**Published:** 2024-09-16

**Authors:** Maurício Figueira, Bianca Schindler, Andréa Onofre de Araujo, Alain Chautems, Mathieu Perret, Nílber Gonçalves da Silva, Ruy José Válka Alves, Marcelo Fragomeni Simon

**Affiliations:** 1 Programa de Pós-graduação em Botânica, Departamento de Botânica, Universidade de Brasília, 70919-970, Brasília, DF, Brazil Universidade de Brasília Brasília Brazil; 2 Universidade Federal de São Carlos, Rodovia João Leme dos Santos (SP-264), km 110, Itinga, 18052-780, Sorocaba SP, Brazil Universidade Federal de São Carlos Sorocaba Brazil; 3 Conservatoire & Jardin botaniques de Genève, 1292 Chambésy, Switzerland Conservatoire et Jardin botaniques (CJB) Geneva Switzerland; 4 Department of Plant Sciences, University of Geneva, 1292 Chambésy, Switzerland University of Geneva Chambésy Switzerland; 5 Universidade Federal do Rio de Janeiro, Museu Nacional, Departamento de Botânica. Quinta da Boa Vista s/n, 20940-040, Rio de Janeiro, RJ, Brazil Universidade Federal do Rio de Janeiro Rio de Janeiro Brazil; 6 Embrapa Recursos Genéticos e Biotecnologia, Parque Estação Biológica, 70770-917, Brasília, DF, Brazil Embrapa Recursos Geneticos e Biotecnologia Brasília Brazil

**Keywords:** Cerrado, Gloxiniinae, protected area, savanna

## Abstract

*Tapinavillosa* (Gesneriaceae) was published by George Gardner in 1842, based on material he collected in Serra de Natividade (Tocantins, Brazil) in 1840. The species is now recognized as *Goyaziavillosa* (Gardner) R.A. Howard. Since Gardner’s travels in Central Brazil, this species had not been collected again and the taxon was considered as possibly extinct. After a long time, we report the discovery of two new populations of *G.villosa* in the municipality of Palmas and in the Estação Ecológica Serra Geral do Tocantins, ca. 200 and 100 km north of the Serra de Natividade, respectively. The newly collected materials allow us to better characterize the morphology and infer the phylogenetic placement of this poorly-known species. Here we demonstrate that *G.villosa* is closely related to *G.rupicola* in a clade including *Goyazia* and *Mandirola* species, and provide an updated description of the species, including field images, photographic plate, information on its distribution and habitat, and a taxonomic key for the species of *Goyazia*.

## ﻿Introduction

**Figure 1.**
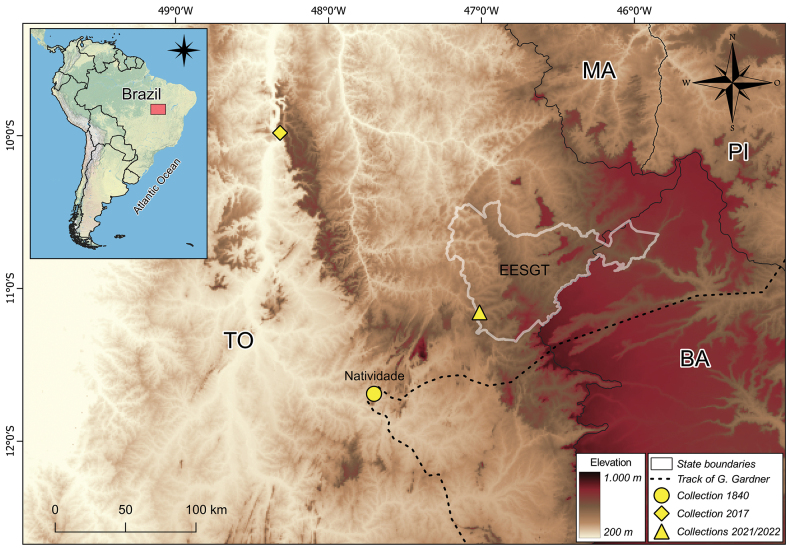
Location of the type collection of *Goyaziavillosa* made in 1840 (circle) and new records made in the municipality of Palmas in 2017 (diamond) and Estação Ecológica Serra Geral do Tocantins (EESGT) in 2021 and 2022 (triangle). Brazilian states: BA (Bahia), MA (Maranhão), PI (Piauí), TO (Tocantins). Track of G. Gardner based on the Atlas dos Viajantes no Brasil (https://viajantes.bbm.usp.br/). Map produced with QGIS (QGIS Development Team 2024).

*George Gardner* (1812–1849), a Scottish botanist, intensively explored the Brazilian Cerrado between the years 1836 and 1841, collecting thousands of plant specimens. He also described and published many new plant species from this phytogeographic region, most of them published in the illustrated botanical magazine named Icones Plantarum (Gardner 1842). Between October 1839 and February 1840, Gardner stayed at Vila de Natividade, a locality today known as the county town Natividade in Tocantins State (Fig. 1), to explore and describe the flora and geology of the mountains east of the town (Gardner 1942: 283). It is where Gardner collected plant specimens that he later described as *Tapinavillosa* Gardner. This species is currently classified as *Goyaziavillosa* (Gardner) R.A. Howard and was until now only known by Gardner’s type collection.

Since the first description of *Tapinavillosa*, this species was subsequently classified into different genera: *Tapeinotesvillosa* (Gardner) Walp., *Ligeriavillosa* (Gardner) Hanst., *Anetanthusvillosus* (Gardner) Benth. & Hook.f. ex B.D. Jacks., and *Gloxiniavillosa* (Gardner) Wiehler. The latter is an illegitimate name because *Gloxiniavillosa* (Gardner) Wiehler is a later homonym with *Gloxiniavillosa* (Lindl.) Mart., which is currently placed in *Sinningiavillosa* Lindl. The current classification of *Tapinavillosa* in *Goyazia* Taubert was proposed by Howard (1975), based on characters such as “shape and lobing of the corolla, adherence of all four anthers, pubescent ovary, and the scaly rhizomes”. The genus *Goyazia* also includes two other species endemic to Brazil (*G.rupicola* Taub. and *G.petraea* (S.M. Phillips) Wiehler) that are also found on rocky outcrops within the Cerrado phytogeographic domain (Araujo 2007, 2024). The genus belongs to the subtribe Gloxiniinae and has strong morphological and phylogenetic affinities with *Mandirola* Decne. (Araújo et al. 2010). Currently, *Goyaziavillosa* is an accepted name in the Flora e Funga do Brasil (Araujo 2024), although Roalson et al. (2005) treated the species as *incertae sedis* and possibly related to *Phinaea* Benth. Therefore, the taxonomic placement of *G.villosa* was ambiguous and needed to be reexamined based on additional morphological and phylogenetic data.

Recent floristic exploration in the savannas of the Jalapão region (Tocantins) has resulted in a large number of new species described, as well as new records of poorly known taxa (e.g. Yamamoto et al. 2008; Araújo et al. 2016; Borges and Antar 2016; Mendes et al. 2017; Antar et al. 2018; Pastore and Antar 2021; Santana and Simon 2022). However, most of this vast area remains poorly collected (Antar and Sano 2019; Santana and Simon 2022). To fill gaps in floristic knowledge, we organized expeditions to different localities in the state of Tocantins in 2017, and also carried out a survey of the vascular flora of the Estação Ecológica Serra Geral do Tocantins (EESGT) between 2018 and 2022. Several floristic novelties that resulted from these expeditions have already been published (Alves et al. 2018; Guimarães et al. 2021; Mendes et al. 2022; Schindler et al. 2023).

In the context of these expeditions, we discovered two populations of *G.villosa* in the central and eastern parts of Tocantins, more than a century and a half after Gardner first collected this species. In this paper, we present an updated and detailed morphological description of *G.villosa* including field images, distribution maps, habitat preferences, as well as a taxonomic key for the species of *Goyazia*. Phylogenetic analyses were also conducted using newly obtained nrDNA ITS sequences to verify the placement of *G.villosa* within the Gloxiniinae.

## ﻿Material and methods

In 2005 and 2013, expeditions aiming to collect *G.villosa* were carried out in the type locality at Serra de Natividade and surrounding areas by two of the authors (AOA and AC). However, no plants of *G.villosa* were located in this area. Subsequent field expeditions were carried out in different parts of the state of Tocantins in 2017. Field expeditions to survey the flora of EESGT were conducted by three of the authors (BS, MF, and MFS) between 2018 and 2022 (Schindler et al. 2023). EESGT is a large protected area (716,306 hectares) located in the eastern portion of the state of Tocantins and the extreme west of the state of Bahia (Fig. 1). According to Köppen’s classification, the climate corresponds to a tropical climate with dry winter, with rain from November to April and marked dry season from May to October (*Aw*) (Alvares et al. 2014), and altitudes range from 400 to 730 m. Vegetation types in EESGT include savannas and grasslands, which dominate the landscape, as well as riverine forests and swamps along water courses (Franke et al. 2018).

Observations and photographic records of the species were carried out in the field in 2017, 2021, and 2022. Collections were deposited in the CEN, G, R, RB, and SORO herbaria (acronyms according to http://sweetgum.nybg.org/science/ih/). Identification of the specimens collected was based on the protologue (Gardner 1842) and comparisons with digital images of the types deposited at BM, CGE, E, FI, G, K, P, and W. Morphological terminology used in the description follows Beentje (2010) and Font Quer (2001) for general morphology, and Ellis et al. (2009) for leaf venation. The assessment of the species conservation status followed the IUCN guidelines (IUCN 2024) using criterion B (geographic range). Estimates of Area of Occupancy (AOO) and Extent of Occurrence (EOO) were calculated using QGIS, version 3.28 (QGIS Development Team 2024). We used land cover maps from the Mapbiomas platform (Projeto MapBiomas, 2024) to estimate *G.villosa* habitat availability and reduction based on the variation of native vegetation and anthropic areas within the EOO of the species between 1986 and 2021.

The ribosomal DNA internal transcribed spacer (ITS) of a newly collected sample of *G.villosa* (*B. Schindler et al.41*) was sequenced to estimate the phylogenetic placement of this species within the Gloxiniinae. Leaves from a plant collected in the field were immediately dried in silica gel. DNA was extracted from tissues samples using the SILEX method (Vilanova et al. 2020). Amplification and sequencing of ITS follow the procedures described in Araújo et al. (2010). The newly acquired sequence was deposited in EMBL/GenBank (no PP468351) and was added to the ITS alignment generated by Araújo et al. (2010), which includes 13 Gloxiniinae genera. Maximum likelihood analysis of this dataset was conducted using the software RAxML (Stamatakis 2014) through the web-server of the Swiss Institute of Bioinformatics (Kozlov et al. 2019). The unpartitioned sequence alignment was analyzed using the GTR + GAMMA model. The robustness of the tree was evaluated with bootstrap resampling and 1000 replicates.

## ﻿Results

### ﻿Taxonomic treatment

#### ﻿ Goyazia villosa

Taxon classificationPlantaeLamialesGesneriaceae

(Gardner) R.A. Howard, J. Arnold Arbor. 56(3): 367. 1975.

B47757F3-7AAA-5BB4-B5C0-AFA18DC9FF20

≡ Tapinavillosa Gardner. Type: Brazil. Goyaz [Tocantins]: in dry clefts of rocks near the summit of the Serra de Natividade, February 1840, *G. Gardner 3875* (lectotype: K [barcode] K000509791 image!; isolectotypes BM [barcodes] BM000793292, BM000883802 images!, CGE [barcode] CGE00055228 image!, E [barcodes] E00062346, E00062347 images!, FI [barcode] FI009756 image!, G [barcodes] G00365532, G00365534, G00365541 images!, P [barcodes] P00587409, P00587410, P00587411 images!, W [barcodes] W0005013, W0192283 images!).

##### Description.

Herb, rupicolous, with a perennial scaly rhizome at the base of the roots, stem 1–10 cm long, erect, unbranched, green *in vivo*, villous. Leaves in basal pseudo-rosette or opposite and arranged in 2–5 pairs along the stem, anisophyllous, internodes 0.2–3 cm long, petiole 2–8 mm long, terete, green, villous, blade 1–3.5 × 0.3–2.5 cm, ovate-elliptic or elliptic, symmetrical, membranaceous, pubescent on both faces, tector trichomes unicellular or multicellular uniseriate, base obtuse or cuneate, apex acute or acuminate, margin serrate with 5–14 teeth, lateral veins 4–5 per side. Inflorescence composed of a single flower on leaf axil, pedicel 1.8–3.5 cm long, erect, villous; sepals subequal, 3–4 × 0.5 mm, linear-lanceolate, subulate at the apex, villous; corolla tubular-infundibuliform, pseudo-actinomorphic, 10–12 mm long, white at base, tube dark purple, lobes 5, 3–4 mm long, subequal, marked with 3 lines of purple and faint dots, with white towards the apex, margin subdentate, throat with wine red dots; stamens 4, filaments glabrous, anthers coherent, nectary dehiscent, annular; ovary superior, ovoid, villous, style 3.5–4 mm long, villous at base, glabrous at the apex, stigma stomatomorph. Fruit a dry loculicidal capsule, dehiscent at the apex, seeds black.

##### Additional specimens examined.

Brazil • Tocantins, Palmas, Serra do Lajeado; 09°58'55"S, 48°19'00"W; 295 m, fl.; 5 Apr 2017; *R.J.V. Alves & N.G. Silva 12586* (CEN, R); • Tocantins, Ponte Alta do Tocantins, ESEC Serra Geral do Tocantins, rio das Balsas, Cachoeira da Fumaça; 11°09'22"S, 47°00'43"W; 467 m, fl. and fr.; 2 Mar 2021; *B. Schindler*, *M. Figueira*, *M.F. Simon*, *V.F. Gomes*, *W.B. Silva 41* (CEN, RB); • idem; 11°09'22"S, 47°00'45"W; 465 m, fl.; 15 Mar 2022; *M. Figueira*, *B. Schindler*, *M.F. Simon*, *R.R. Souza*, *V.F. Gomes*, *W.B. Silva 1808* (CEN, G [barcode] G00447873, R, RB, UB).

##### Distribution and habitat.

**Figure 2.**
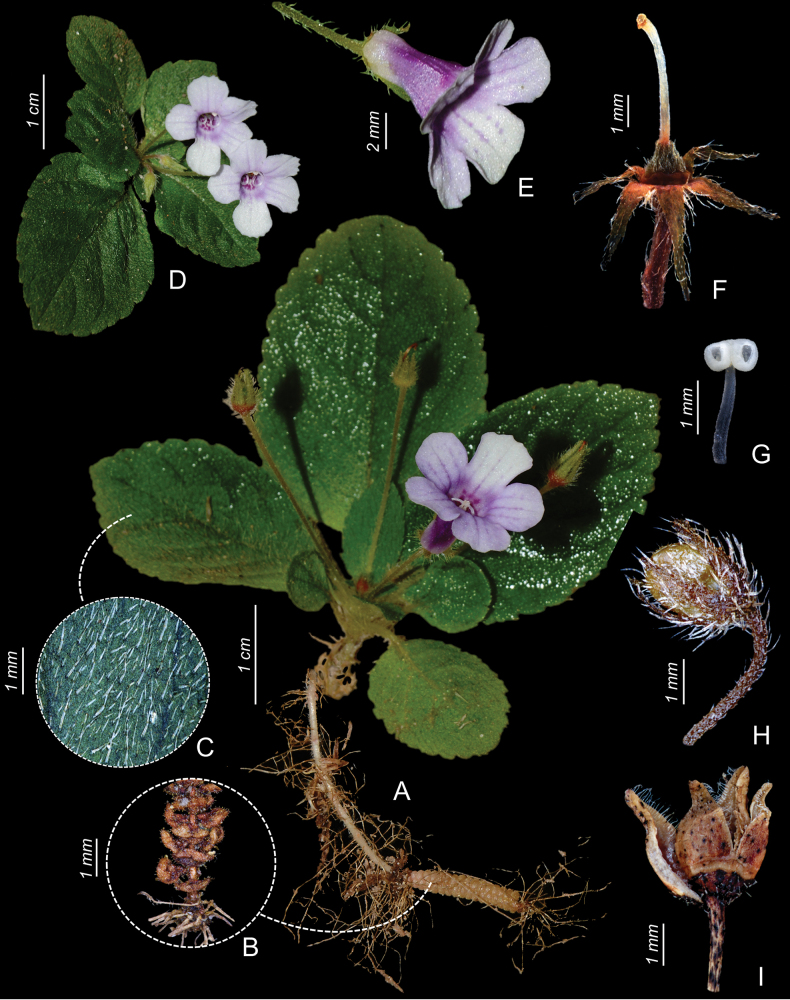
*Goyaziavillosa* (Gardner) R.A. Howard **A** habit **B** detail of rhizome with fleshy scales **C** detail of leaf trichomes on the adaxial surface **D** front view of corolla **E** lateral view of corolla **F** detail of sepals, ovary, and ring nectary (corolla removed) **G** stamen **H** immature fruit **I** open capsule. Photographs by M. Figueira and B. Schindler from M. Figueira et al. 1808.

**Figure 3.**
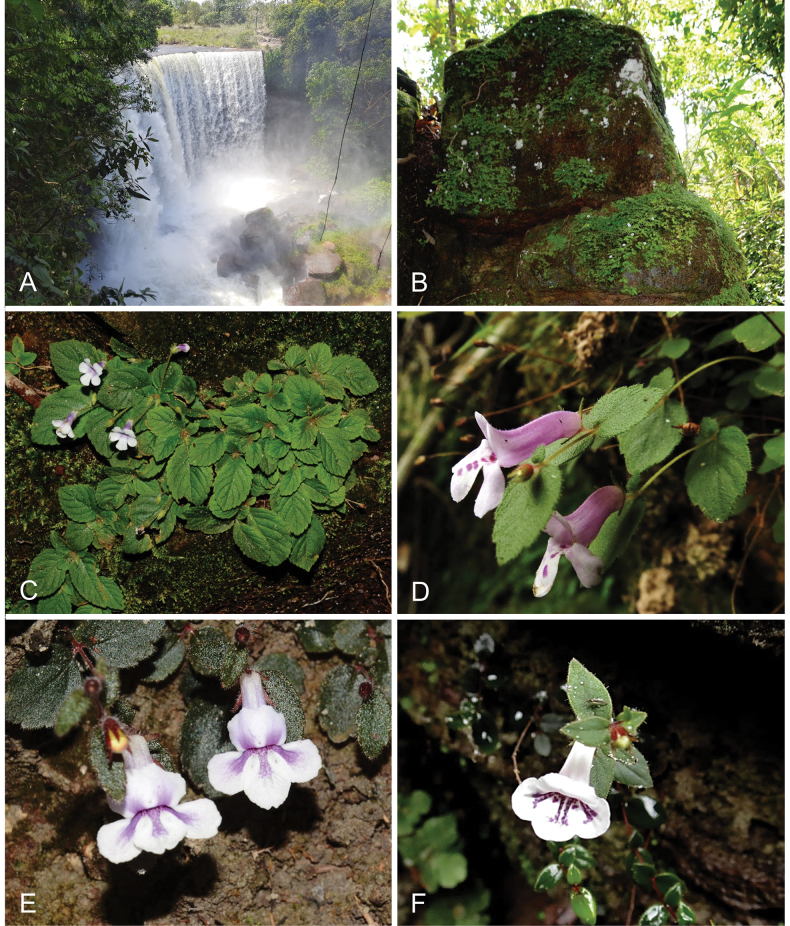
Habitat and habit of *Goyazia***A** Cachoeira da Fumaça at the Estação Ecológica Serra Geral do Tocantins **B** habitat of *G.villosa* on rocky outcrops **C** frontal view of *G.villosa* attached to sandstone rocks **D** lateral view of individuals of *G.petraea***E, F** habit of *G.rupicola*. Photographs: **A–C**, **E** M. Figueira and B. Schindler **D** and **F** A.O. Araujo; photos from collections: **B, C** M. Figueira et al. 1808 **D** A.O. Araujo et al. 1065-24 **E** M. Figueira et al. 1897 **F** A.O. Araujo et al. 1082.

The species is endemic to the state of Tocantins, Brazil (Fig. 1). It has been recorded in the municipalities of Natividade, Palmas, and Ponte Alta do Tocantins. Recent records were made in a humid and shaded hillside, in the Serra do Lajeado, at around 295 m of altitude (*R.J.V. Alves & N.G. Silva 12586*), and also in a riparian forest on shaded humid sandstone walls in the locality known as Cachoeira da Fumaça, in the southwest corner of the EESGT, at around 470 m of altitude (*B. Schindler et al. 41 and M. Figueira et al. 1808*) (Fig. 3A–C). Description of the habitat by Gardner on the specimen label indicates that the plant was collected “in dry clefts of rocks near the summit of the Serra de Natividade”. In contrast, our field observations suggest that *Goyaziavillosa* grows in humid and shaded sites. Preference of *G.villosa* for drier sites is however not unlikely because of its rhizomes with fleshy scales (Fig. 2B) that allow the plant to become dormant during the dry season, and grow and reproduce during the rainy season. This feature has already been reported for other genera of Gloxiniinae (Araújo et al. 2010).

##### Phenology.

Flowers and fruits between December and April during the rainy season.

##### Etymology.

The genus name is a reference to the state of Goiás (formerly spelled as Goyaz in old Portuguese orthography). The plant was collected in an area that now belongs to the state of Tocantins. The specific epithet refers to the trichomes found throughout the plant.

##### Conservation status assessment.

*Goyaziavillosa* is currently known from only three localities, where it inhabits a very specific habitat. It has an AOO of 12 km^2^ and EOO 9103.44 km^2^. Analysis of land use within *G.villosa* range (EOO) revealed a reduction of 1616 km^2^ over 35 years, which represents an increment of anthropic areas from 9.0% in 1986 to 17.7% in 2021. Considering its AOO < 500 km^2^, number of locations ≤5, and continuing decline in extent of occurrence, we preliminary assign a category of endangered (EN) for *G.villosa* under IUCN criterion B2ab(i).

Although known from only three localities and inhabiting a very specific habitat, *G.villosa* occurs in a region that still harbors much of its native vegetation, despite the observed vegetation loss over its range. The two newly reported populations are between 100 and 200 km from the type collection. The area covered within its current range harbors a number of unexplored sites of potential occurrence of *G.villosa*, including several mountain ranges, which could increase its AOO. Recently documented habitats of *G.villosa* are either unsuitable for agriculture (rocky outcrops) or protected by environmental regulations (riparian forests), which reduces the chances of loss of this specific habitat. The overall extent of occurrence of *G.villosa* in eastern Tocantins includes a large protected area (EESGT). One negative aspect in the conservation of this species is that the population found in Cachoeira da Fumaça within the EESGT may suffer from trampling since a patch of individuals of *G.villosa* is located along a trail used by the numerous visitors to the waterfall. The rarity of *G.villosa* is probably a result of strong habitat specificity and insufficient collecting effort across its geographic range, which could explain the paucity of records. Finally, more sampling is needed to understand its entire geographical range.

### ﻿Updated key to species of *Goyazia*

**Table:** 

1	Plant rosette-like; stems not-filiform, villose; leaf margin usually bearing more than 5 teeth per side, petioles 2–8 mm long; flowers long-pedicellate, pedicel more than 1.8 cm long, corolla tubular-infundibuliform, pseudo-actinomorphic, macules restricted to the throat with lines of faint dots on the lobes (endemic to the state of Tocantins)	***G.villosa* (Figs 2, 3B, C)**
–	Plant with developed stem; stems filiform, glabrous or pubescent; leaf margin entire or bearing 2 or 3 teeth per side, petioles inconspicuous or lacking; flowers short-pedicellate, pedicel less than 0.5 cm long, corolla bilabiate, zygomorphic, macules spreading to the lobes (widespread in the Brazilian Cerrado)	**2**
2	Corolla tube externally white, light lilac, or with purplish to lilac longitudinal lines; nectary forming a ring (occurring in the states of Goiás, Maranhão, Minas Gerais, Pará, and Tocantins)	***G.rupicola* (Fig. 3E, F)**
–	Corolla tube externally pink or lilac; nectary formed by 5 glands, lobed and free from each other (occurring in the states of Mato Grosso and Mato Grosso do Sul)	***G.petraea* (Fig. 3D)**

### ﻿Phylogenetic relationships

**Figure 4.**
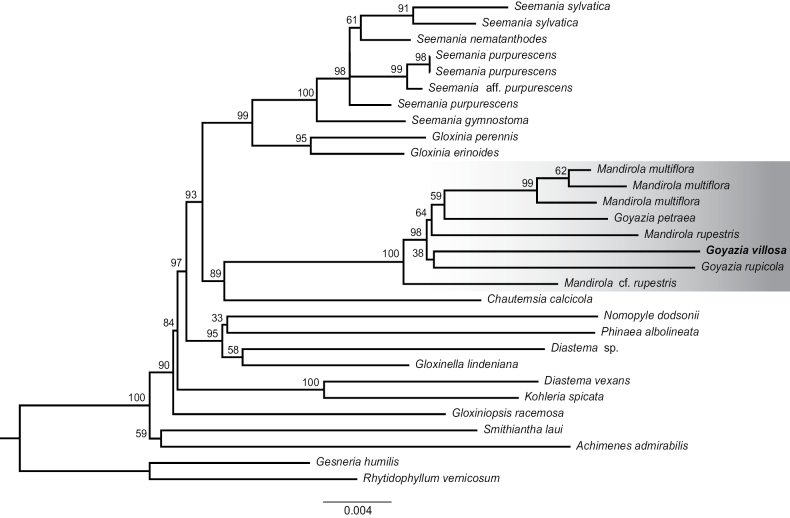
Maximum Likelihood tree resulting from the analysis of the nuclear ribosomal internal transcribed spacer (ITS) of 28 accessions of subtribe Gloxiniinae and two outgroups from subtribe Gesneriinae. The clade containing the rediscovered *Goyaziavillosa* is highlighted in gray. Numbers above branches are bootstrap support values.

The topology of the phylogenetic tree resulting from our RAxML analysis (Fig. 4) is fully congruent with the Bayesian 50% majority rule consensus tree shown in Araújo et al. (2010). *G.villosa* is inferred as sister to *G.rupicola* but this relationship is not statistically supported (BS < 50%). In contrast, *G.villosa* is nested in a strongly supported clade (BS = 100%) that includes all *Goyazia* and *Mandirola* species.

## ﻿Discussion

The rediscovery of *G.villosa* was made possible by active search and botanical exploration of moist cliffs, and its very specialized habitat. These cliffs are known for hosting vascular plant assemblages, which typically include small species of Gesneriaceae, *Pitcairnia* (Bromeliaceae), *Anemia* (Anemiaceae), and liverworts. Gardner’s description of the locality of *G.villosa* as ‘dry’ may refer to the rocky clefts providing protection from direct insolation, thereby maintaining a relatively humid microclimate. The populations rediscovered between 2017 and 2022 were growing in very humid conditions. These occurrences confirm that the species still persists in the wild and calls for more intensive field surveys of the Cerrado’s endemic flora. Analysis of the recent collections of *G.villosa* allowed a better understanding of the morphological traits that characterize this species. The presence of a scaly rhizome is illustrated here for the first time (Fig. 2B). This feature, together with the small habit of the plant, the coherent four anthers, and the pubescent ovary are congruent with the placement of this species in the genus *Goyazia* (Howard 1975). The species can be easily differentiated from the two congeners (*G.petraea* and *G.rupicola*) by serrate leaf margin and long (1.8–3.5 cm) pedicels (Figs 2, 3D).

Recent collections also provided material for ongoing studies on pollen micromorphology that preliminarily show that *G.villosa* shares many characters with the two other species of *Goyazia* and the three species of *Mandirola* (Souza et al., in prep.). In agreement with morphology, our preliminary phylogenetic analysis based on a single nuclear marker (ITS) places *G.villosa* together with *G.rupicola*, the type species of the genus, in a well-supported clade (BS = 100%) including species of *Goyazia* and *Mandirola* (Fig. 4). A natural intergeneric hybrid was recently described under the name *Goydirola* A.O. Araujo & M. Peixoto, indicating the close relationships between the two genera (Araujo et al. 2021). However, a more thorough study of the phylogenetic relationships based on a larger number of genes (Ogutcen et al. 2021) and a larger infraspecific sampling (Fiorini et al. 2020) is still needed to clarify the generic circumscription of *Goyazia* and *Mandirola* and to define the closest relative species of *G.villosa*.

## Supplementary Material

XML Treatment for Goyazia villosa

## ﻿Additional information

### Author ORCIDs

Maurício Figueira https://orcid.org/0000-0002-5770-7002

Bianca Schindler https://orcid.org/0000-0002-4719-0601

Andréa Onofre de Araujo https://orcid.org/0000-0002-7968-1595

Alain Chautems https://orcid.org/0000-0002-0086-1623

Mathieu Perret https://orcid.org/0000-0003-2021-114X

Nílber Gonçalves da Silva https://orcid.org/0000-0002-2179-1898

Ruy José Válka Alves https://orcid.org/0000-0001-5123-9124

Marcelo Fragomeni Simon https://orcid.org/0000-0002-5732-1716

### Data availability

All of the data that support the findings of this study are available in the main text.
